# LIGHT (TNFSF14) Increases the Survival and Proliferation of Human Bone Marrow-Derived Mesenchymal Stem Cells

**DOI:** 10.1371/journal.pone.0166589

**Published:** 2016-11-11

**Authors:** Sook-Kyoung Heo, Eui-Kyu Noh, Gi-Dong Gwon, Jeong Yi Kim, Jae-Cheol Jo, Yunsuk Choi, SuJin Koh, Jin Ho Baek, Young Joo Min, Hawk Kim

**Affiliations:** 1 Biomedical Research Center, Ulsan University Hospital, University of Ulsan College of Medicine, Ulsan 682-060, Republic of Korea; 2 Department of Hematology and Oncology, Ulsan University Hospital, University of Ulsan College of Medicine, Ulsan 682-714, Republic of Korea; Instituto Butantan, BRAZIL

## Abstract

LIGHT (HVEM-L, TNFSF14, or CD258), an entity homologous to lymphotoxins, with inducible nature and the ability to compete with herpes simplex virus glycoprotein D for herpes virus entry mediator (HVEM)/tumor necrosis factor (TNF)-related 2, is a member of the TNF superfamily. It is expressed as a homotrimer on activated T cells and dendritic cells (DCs), and has three receptors: HVEM, LT-β receptor (LTβR), and decoy receptor 3 (DcR3). So far, three receptors with distinct cellular expression patterns are known to interact with LIGHT. Follicular DCs and stromal cells bind LIGHT through LTβR. We monitored the effects of LIGHT on human bone marrow-derived mesenchymal stem cells (BM-MSCs). At first, we checked the negative and positive differentiation markers of BM-MSCs. And we confirmed the quality of MSCs by staining cells undergoing adipogenesis (Oil Red O staining), chondrogenesis (Alcian blue staining), and osteogenesis (Alizarin red staining). After rhLIGHT treatment, we monitored the count, viability, and proliferation of cells and cell cycle distribution. PDGF and TGFβ production by rhLIGHT was examined by ELISA, and the underlying biological mechanisms were studied by immunoblotting by rhLIGHT treatment. LTβR was constitutively expressed on the surface of human BM-MSCs. Cell number and viability increased after rhLIGHT treatment. BM-MSC proliferation was induced by an increase in the S/G_2_/M phase. The expression of not only diverse cyclins such as cyclin B1, D1, D3, and E, but also CDK1 and CDK2, increased, while that of p27 decreased, after rhLIGHT treatment. RhLIGHT-induced PDGF and TGFβ production mediated by STAT3 and Smad3 activation accelerated BM-MSC proliferation. Thus, LIGHT and LTβR interaction increases the survival and proliferation of human BM-MSCs, and therefore, LIGHT might play an important role in stem cell therapy.

## Introduction

Mesenchymal stem cells (MSCs), a type of adult stem cells, are self-renewing, multipotent cells capable of differentiating into multiple cell types such as adipocytes, chondrocytes, and osteocytes [1–3]. They can be found in many tissues such as the bone marrow (BM), skeletal muscle, dental pulp, bone, umbilical cord, and adipose tissue [2,4].

MSCs are of great interest in the areas of regenerative medicine and immunotherapy because of their unique biological properties and diverse properties, including differentiation, homing, and trophic function [5]. In particular, MSCs showed great potential for the replacement of damaged tissues such as bone, cartilage, and tendon [6]. In addition, MSCs possess immunomodulatory properties that can modulate immune as well as inflammatory responses [4,7–9]. MSCs have therapeutic potential in diseases such as osteogenesis imperfecta [10], graft-versus-host disease (GVHD) [11–13], myocardial infarction [14,15], Crohn’s disease [16], alcoholic cirrhosis [17], and amyotrophic lateral sclerosis [18,19]. Many studies affirm the effectiveness of these treatments. However, only low cell numbers (1–10 of 1 × 10^5^ nucleated cells) have been obtained from healthy volunteers by BM aspiration [7]. Thus, clinical application has suffered because of limitations such as low cell number. Therefore, it is necessary to search for alternative methods.

The interaction between tumor necrosis factor (TNF) and TNF receptor (TNFR) plays important roles in cell differentiation, survival, and death, which further orchestrates lymphoid organogenesis, activation, and homeostasis of immune cells [20,21]. LIGHT (HVEM-L, TNFSF14, or CD258), an entity homologous to lymphotoxins, with inducible nature, and able to compete with herpes simplex virus glycoprotein D for herpes virus entry mediator (HVEM)/tumor necrosis factor (TNF)-related 2 is a member of the TNF superfamily [22,23]. It is a 29-kDa type II transmembrane protein, is expressed as a homotrimer on activated T cells as well as DCs, and has three receptors, namely, HVEM, LT-β receptor (LTβR, TNFRSF3) and decoy receptor 3 (DcR3) [20,22]. So far, three receptors with distinct cellular expression patterns have been known to interact with LIGHT [24–26]: HVEM (TNFRSF14, CD270) detected on activated DCs, T and B cells, NK cells, monocytes, and endothelial cells [26–28]; LTβR found on follicular DCs and stromal cells and binds LIGHT [25]; and the soluble entity decoy receptor 3 (DcR3) detected on diverse cancer cells such as multiple myeloma and diffuse large B-cell lymphoma [29–31]. Moreover, the serologic DcR3 levels are associated with advanced liver diseases [32].

To date, LIGHT and HVEM interaction leading to T cell activation [26,28], and lymphotoxin α/β and LTβR interaction contributes to the organization of lymphoid architecture and cellular positioning [25]. However, the effects of LIGHT in human BM-MSCs are unclear. Therefore, we monitored the roles of LIGHT and LTβR interaction in human BM-MSCs and studied the underlying intracellular mechanism.

## Materials and Methods

### Reagents

Recombinant human LIGHT (rhLIGHT) was purchased from R&D Systems (Minneapolis, MN), and diluted in 0.1% BSA-PBS buffer. The CellTiter 96 AQueous One Solution Cell Proliferation Assay (MTS) was purchased from Promega (Madison, WI, USA). StemPro^®^ MSC SFM CTS^™^, StemPro^®^ Adipogenesis Differentiation Kit, StemPro^®^ Chondrogenesis Differentiation Kit, StemPro^®^ Osteogenesis Differentiation Kit, and fetal bovine serum (FBS) were obtained from GibcoBRL (Grand Island, NY, USA). Oil Red O staining kit (for adipocytes), Alcian blue staining kit (for chondrocytes) and Alizarin red staining (for osteocytes) were purchased from Invitrogen (Camarillo, CA, USA). The antibodies for western blotting, namely, anti-CDK2, cyclin E, and β-actin, were purchased from Santa Cruz Biotechnology (Santa Cruz, CA, USA). Anti-p27, p-STAT3, p-Smad3, STAT3, Smad3, CDK1, cyclin B1, cyclin D1, and cyclin D3 were purchased from Cell Signaling Technology (Beverly, MA, USA). Anti-HVEM, LTβR, CD19, CD34, CD45, CD44, CD90, and CD105 antibodies and PI/RNase solution were purchased from BD Bioscience (San Jose, CA, USA). ELISA for PDGF-BB and TGF-β1 were purchased from R&D Systems. The Cell Proliferation ELISA, BrdU Assay Kit was purchased from Roche Diagnostics (San Francisco, CA, USA). All reagents were obtained from Sigma-Aldrich (St. Louis, MO, USA).

### Human samples

Blood (n = 4) and BM samples (n = 4) were collected once from all healthy volunteers participating in this study at the Ulsan University Hospital, Ulsan, South Korea.

### Ethics statement

All subjects provided informed written consent. The study protocol was approved by the Ulsan University Hospital Institutional Review Board (UUH-IRB-2016-07-026).

### BM-derived MSC isolation and culture

Mononuclear cells (MNCs) were isolated from the BM suspension by gradient centrifugation with Lymphoprep (Axis-Shield, Oslo, Norway; density, 1.077 g/mL) and loaded into 100-mm culture dishes containing DMEM (low glucose) with 10% FBS and 1% penicillin and streptomycin. The most common method is based on the capacity of MSCs to adhere to plastic surfaces [2,33]. After 3-day culture in a humidified incubator at 37°C and 5% CO_2_, the non-adhering cells were washed from the flask using PBS. Adherent cells were grown to reach confluence and passaged. After two passages, the cells were cryopreserved in FBS with 10% DMSO. The MSCs used throughout this study were between passage 2 and 5. BM-MSCs were maintained in MSC basal medium, namely, StemPro^®^ MSC SFM CTS^™^.

### Flow cytometric phenotypic analysis

Cells were harvested and washed twice with FACS buffer (PBS containing 0.3% BSA and 0.1% NaN3). Cells were incubated with diverse antibodies against each cell surface antigen such as HVEM, LTβR, CD19, CD34, CD45, CD44, CD90, and CD105 (BD Bioscience, San Diego, CA, USA) on ice for 30 min. Cells were then washed twice with FACS buffer and analyzed using the FACSCalibur flow cytometer and CellQuest Pro software (BD Bioscience).

### Differentiation of BM-MSCs into adipocytes, chondrocytes, and osteocytes

BM-MSCs were cultured in specific adipogenic, chondrogenic, and osteogenic differentiation media (GibcoBRL). After 21 days, the cells were harvested and stained by each staining kits. Briefly, lipid droplets were visualized with Oil Red O staining in the adipogenic cultures. In the chondrogenic cultures, cells were stained with Alcian blue. The osteogenic cultures were analyzed for the presence of osteocytes by staining of calcium deposits with Alizarin red (Invitrogen).

### Trypan blue exclusion assay

BM-MSCs were incubated with 0, 100, and 200 ng/mL rhLIGHT for 72 h at 37°C. Cells were inoculated at a density of 4 × 10^4^ cells in each concentration, and grown for 72 h. The grown cells were then harvested, and trypan blue was added to the cell suspension to a final concentration of 0.04%. Cells excluding trypan blue (viable cells) were counted under the microscope with a hemocytometer. Each test was repeated a minimum of four times.

### Cell viability assay (MTS assay)

Cells were seeded in 96-well plates at a density of 2 × 10^4^ cells/mL, with 100 μL of medium per well, and incubated with 0, 100, and 200 ng/mL rhLIGHT for 72 h at 37°C. MTS assay was performed as previously described [34].

### Cell proliferation assay (BrdU assay)

BM-MSCs were incubated with 0, 100, and 200 ng/mL rhLIGHT for 72 h at 37°C. Cell proliferation was measured by a BrdU-(5′-bromo-2-deoxyuridine) enzyme-linked immunosorbent assay (Cell Proliferation ELISA, BrdU; Roche Diagnostics), according to the manufacturer’s instructions. Cells were cultivated under same conditions. For the BrdU assay, the cells were fixed, and their DNA was denatured and blocked, following which the samples were incubated with an anti-BrdU monoclonal antibody coupled to peroxidase and 3,3′,5,5′-tetramethylbenzidine (TMB). Next, absorbance was measured with a PowerWave XS2 Microplate Spectrophotometer (BioTek) at 490 nm. The results are expressed as percentage changes from the basal conditions by using three to five culture wells for each experimental condition.

### Western blotting

Cells were incubated with various concentrations of rhLIGHT for 72 h at 37°C. They were washed three times with ice-cold PBS and then harvested. Western blotting was performed as previously described [34].

### Cytokine ELISA

BM-MSCs were incubated with various concentrations of rhLIGHT for 72 h at 37°C. Cell-free supernatants were collected and frozen at -80°C. Cytokine concentrations were determined using ELISA kits for PDGF-BB and TGF-β1 (R&D Systems).

### Cell cycle analysis by flow cytometry

BM-MSCs were incubated with various concentrations of rhLIGHT for 72 h at 37°C, and then washed with PBS and fixed with 70% ice-cold ethanol for 24 h at 4°C. The fixed cells were rinsed twice with PBS to remove ethanol, and then incubated with 500 ml of PI/RNase A Staining Buffer (cat. No. 550825; BD Bioscience) per test, and incubated for 15 min at room temperature before analysis. Samples were analyzed by FACSCalibur flow cytometer and CellQuest Pro software (BD Bioscience).

### Microarray analysis

BM-MSCs were incubated with 0, 100, and 200 ng/mL rhLIGHT for 48 h, and analyzed using a 44K oligo-microarray (Agilent Technologies, Inc., Palo Alto, CA, USA). Microarray analysis was performed as previously described [34].

### Microarray data analysis

Microarray data analysis was performed as previously described [34]. Expression changes of >2-fold were considered significant. For understanding expression patterns, hierarchical clustering analysis was performed using GeneSpring software. Functional enrichment analyses were performed using the Gene Ontology (GO) functional classification system (www.geneontology.org) or DAVID (http://david.abcc.ncifcrf.gov/) (GEO accession: GSE85895).

### Statistical analysis

The data represent the mean ± standard error of mean (SEM) of a minimum of three independent experiments. All values were evaluated by one-way analysis of variance, followed by Tukey’s range test (GraphPad Prism 6.0). Differences were considered significant at *P* < 0.05.

## Results

### Negative and positive markers are confirmed in BM-derived MSCs

**Fig 1:**
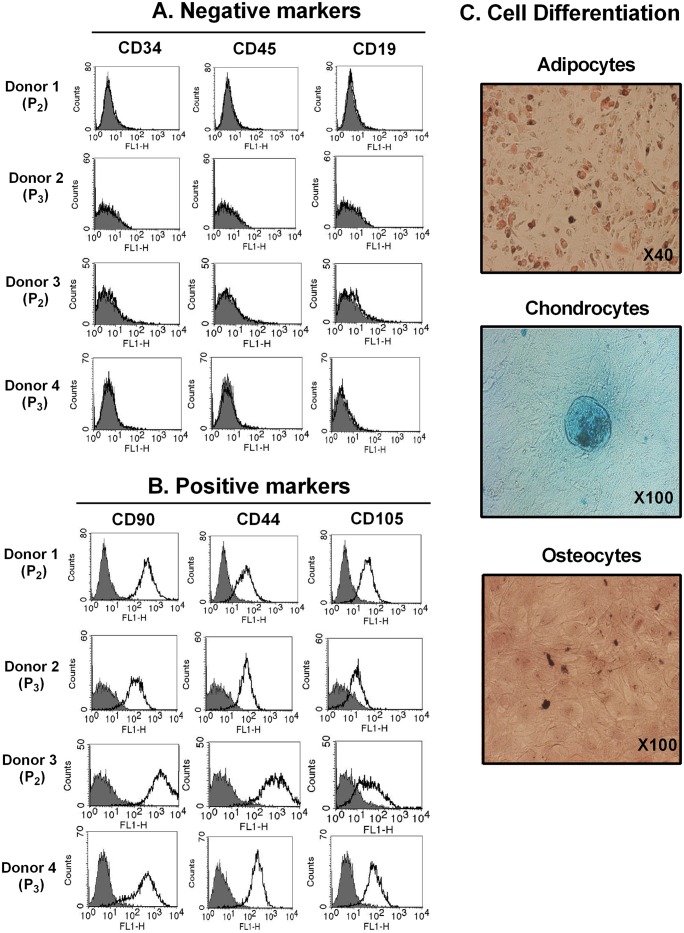
Characterization of bone marrow-derived MSCs (BM-MSCs). (A) Negative marker (CD34, CD45, and CD19) staining in BM-MSCs. (B) Positive marker (CD90, CD44, and CD105) staining in BM-MSCs. The level of expression of each marker was determined by FACS analysis. Filled histogram represents the isotype control (mouse IgG); open histogram represents each antigen. (C) Representative images of cells such as adipocytes, chondrocytes, and osteocytes undergoing differentiation via adipogenesis (Oil Red O staining), chondrogenesis (Alcian blue staining), and osteogenesis (Alizarin red staining), respectively. MSCs, mesenchymal stromal cells, MSCs; BM-MSCs, bone marrow-derived MSCs; P, passage number.

Phenotypic characterization of MSCs is usually performed by FACS analysis of cell surface molecule expression [2,5,6]. Following isolation and subsequent expansion, the phenotype of BM-MSCs were confirmed, including the negative markers (CD34, CD45, and CD19; Fig 1A) and positive markers (CD90, CD44, and CD105; Fig 1B). MSCs are capable of self-renewal and differentiation into multiple cell types, including osteocytes, chondrocytes, and adipocytes [1,7]. We next analyzed the ability of BM-MSCs to differentiate into adipocytes, chondrocytes, and osteoblasts, as shown in Fig 1C. Thus, BM-MSCs were determined phenotypically and their ability to differentiate into mature mesodermal cell types was apparent (Fig 1).

### LT-β Receptor (LTβR) is constitutively expressed in human BM-MSCs

**Fig 2:**
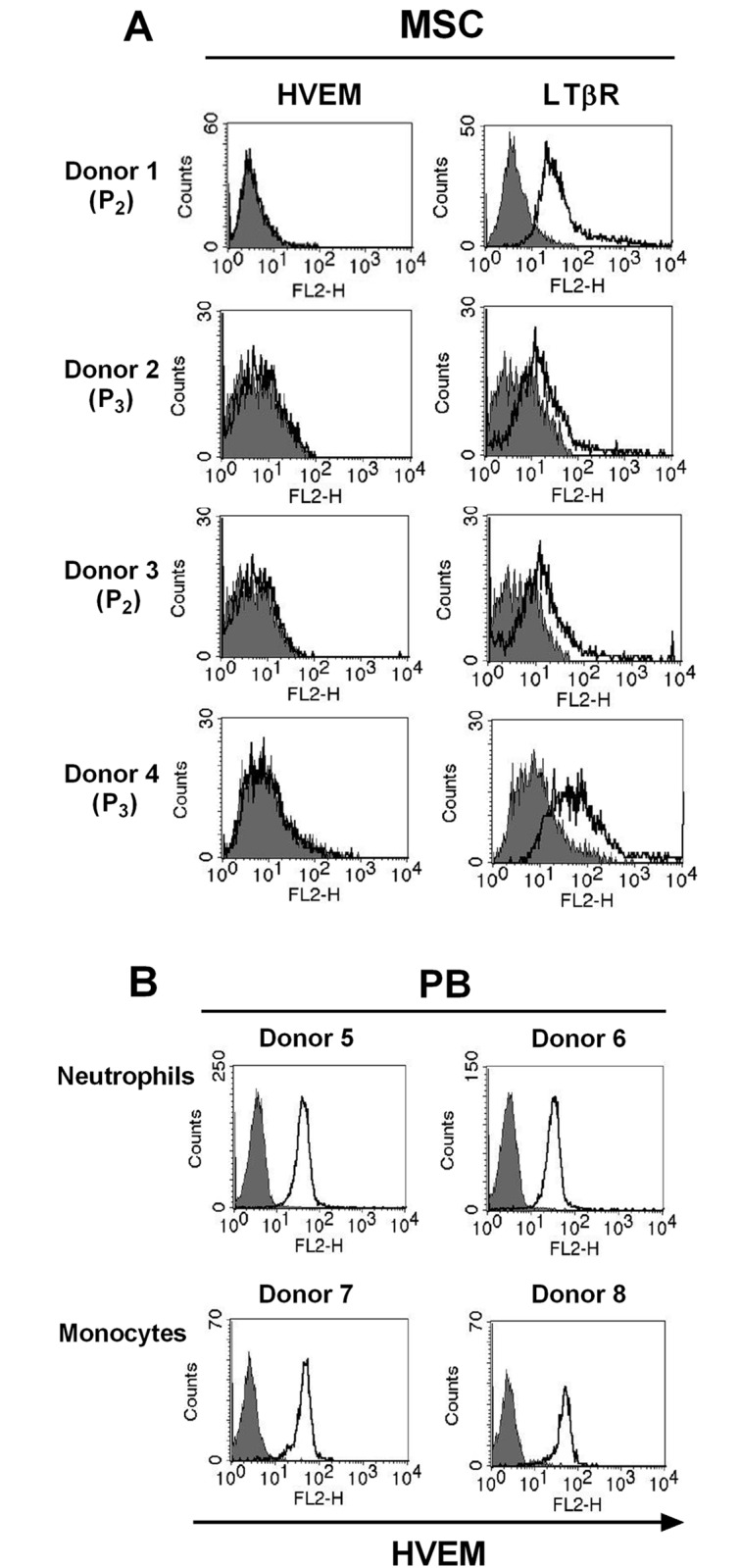
HVEM and LTβR expression in BM-MSCs. (A) HVEM and LTβR on the cell surface of human BM-MSCs were determined by FACS analysis (see Methods). Filled histogram represents the isotype control (mouse IgG); open histogram represents human HVEM or LTβR. (B) HVEMs on the cell surface of human neutrophils and monocytes were determined by FACS analysis. HVEM, herpes virus entry mediator; LTβR, lymphotoxin β receptor; P, passage number.

At first, we examined whether BM-MSCs express LIGHT receptors such as LTβR and HVEM. As shown in Fig 2A, the results of FACS analysis showed that LTβR receptor was expressed in human BM-MSCs, but not HVEM. HVEM are constitutively expressed in human neutrophils and monocytes [26,27], and similar results are shown in Fig 2B. Therefore, these results indicate that LIGHT binds only LTβR in human BM-MSCs.

### LIGHT and LTβR interaction increases cell survival and proliferation in human BM-MSCs

**Fig 3:**
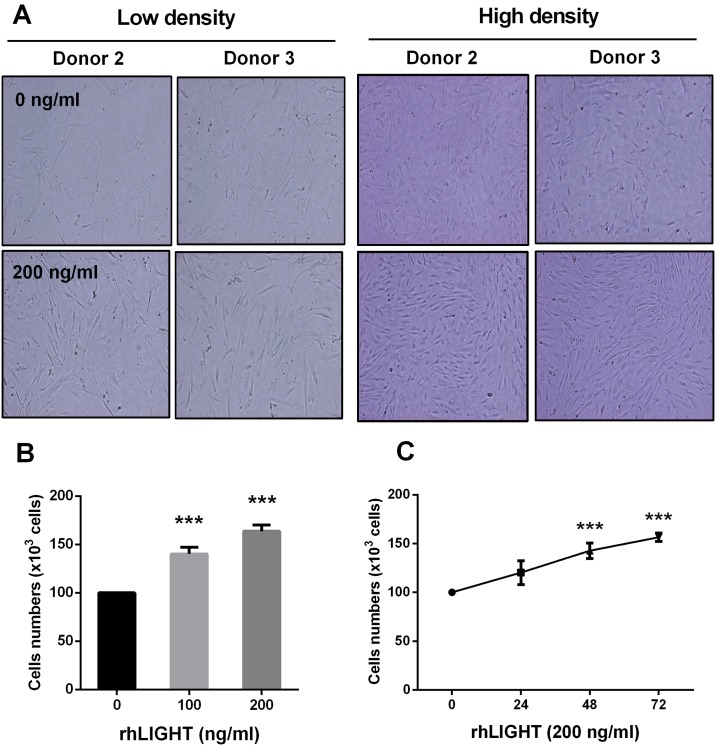
RhLIGHT increases the number of human BM-MSCs. Cells were incubated with 0, 100, and 200 ng/mL rhLIGHT for 72 h. (A) Images of low density (left panel) and high density (right panel) by BSA-control treatment (0.1% BSA-PBS buffer, upper panel) and rhLIGHT treatment (lower panel) in the BM-MSCs. (B) Dose-dependent effect of rhLIGHT on the number of human BM-MSCs at 72 h. (C) Time-dependent effect of rhLIGHT (200 ng/mL) on the number of human BM-MSCs. Data represent the mean ± SEM. Significantly different from the control cells (*); ***, *P* < 0.001. BSA, bovine serum albumin.

We confirmed the effects of rhLIGHT on changes in cell number, survival, and proliferation. RhLIGHT and LTβR interaction increased the number of BM-MSCs, as observed by using an inverted microscope (Fig 3A). BM-MSCs were stimulated with 0, 100, and 200 ng/mL rhLIGHT for 72 h, and the cell count was determined by trypan blue exclusion assay. This effect worked in a dose- and time-dependent manner (Fig 3B and 3C).

**Fig 4:**
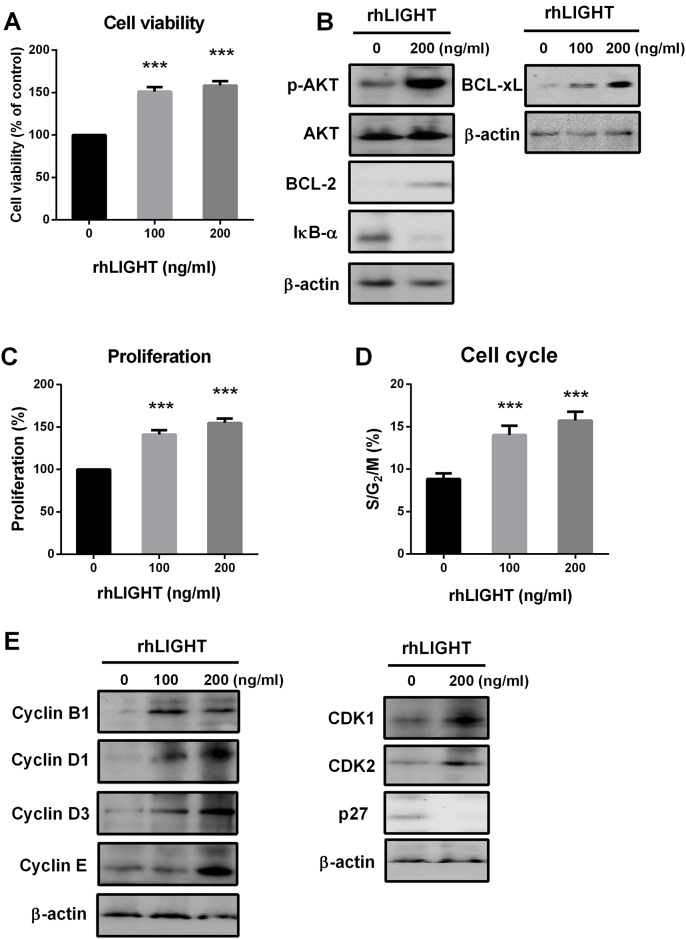
RhLIGHT enhances the viability and proliferation of human BM-MSCs. Cells were incubated with 0, 100, and 200 ng/mL rhLIGHT for 72 h. (A) Cell viability of BM-MSCs, as determined by MTS assay. (B) Expression of survival proteins and anti-apoptotic proteins, as determined by western blotting. (C) Cell proliferation of BM-MSCs, as determined by BrdU assay. (D) Cell cycle distribution of BM-MSCs, as determined by PI/RNase assay (E) Expression of cell cycle-related proteins, as determined by western blotting. The membrane was stripped and reprobed with anti-β-actin mAb to confirm equal loading. Data represent the mean ± SEM. Significantly different from the control cells (*); ***, *P* < 0.001.

Next, we tested the effects of rhLIGHT on cell viability and diverse survival proteins such as AKT, Bcl-2, Bcl-xL, and NF-*k*B. Briefly, not only cell viability, but also the expression of p-AKT, Bcl-2, and Bcl-xL was significantly increased by rhLIGHT treatment of BM-MSCs (Fig 4A and 4B). Moreover, rhLIGHT-induced IkB-α degradation activated NF-kB signal (Fig 4B).

In addition, rhLIGHT increased cell proliferation by increasing the S/G2/M phase in BM-MSCs (Fig 4C and 4D). Cell cycle regulatory proteins were enhanced by rhLIGHT in BM-MSCs, including cyclin B1, D1, D3, and E, and cyclin-dependent kinase (CDK) 1 and 2 (Fig 4E). Furthermore, the expression of the CDK inhibitor, p27, was significantly decreased by rhLIGHT. Thus, LIGHT enhanced cell proliferation by promoting cell cycle and diverse cell cycle regulatory proteins. Therefore, these results indicate that LIGHT enhances cell survival and proliferation in human BM-MSCs via LTβR (Figs 3 and 4).

### LIGHT promotes PDGF and TGF-β production in human BM-MSCs via activation of STAT3 and Smad3

**Fig 5:**
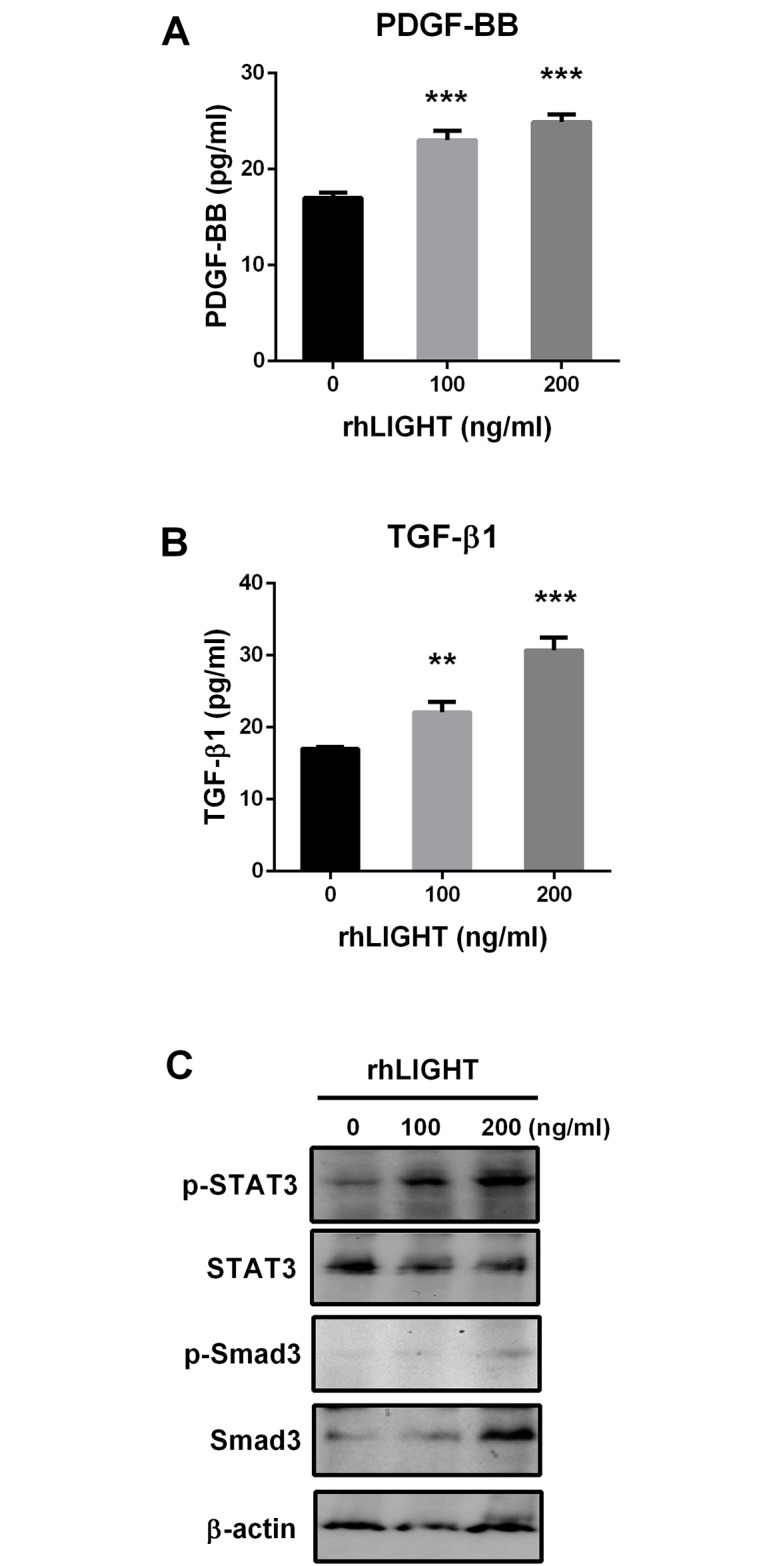
RhLIGHT enhances PDGF-BB and TGF-β production by STAT3 and Smad3 activation in BM-MSCs. Cells were incubated with 0, 100, and 200 ng/mL rhLIGHT for 72 h at 37°C, and the supernatant was collected. (A) PDGF-BB production, as determined by ELISA assay. (B) TGF-β production, as determined by ELISA assay. (C) Expression of p-STAT3, STAT3, p-smad 3, and smad 3, as determined by western blotting. The membrane was stripped and reprobed with anti-β-actin mAb to confirm equal loading. Data represent the mean ± SEM. Significantly different from the control cells (*); **, *P* < 0.01; ***, *P* < 0.001.

PDGF, TGF-β, and FGF signaling are important for the differentiation and growth of MSCs [35]. We measured PDGF and TGF-β production. BM-MSCs were incubated with various concentrations of rhLIGHT for 72 h at 37°C. Cell-free supernatants were collected, and PDGF-BB and TGF-β1 were assayed using ELISA kits. Interestingly, rhLIGHT induced PDGF-BB and TGF-β1 production in BM-MSCs, as shown in Fig 5A and 5B. The expression of p-STAT3, p-Smad3, and Smad3 was dramatically increased by rhLIGHT at 72 h (Fig 5C). Therefore, these results indicate that LIGHT promotes PDGF and TGF-β production in human BM-MSCs via activation of STAT3 and Smad3 (Fig 5).

### LIGHT upregulates the genes for TNF and chemokines in BM-MSCs

**Fig 6:**
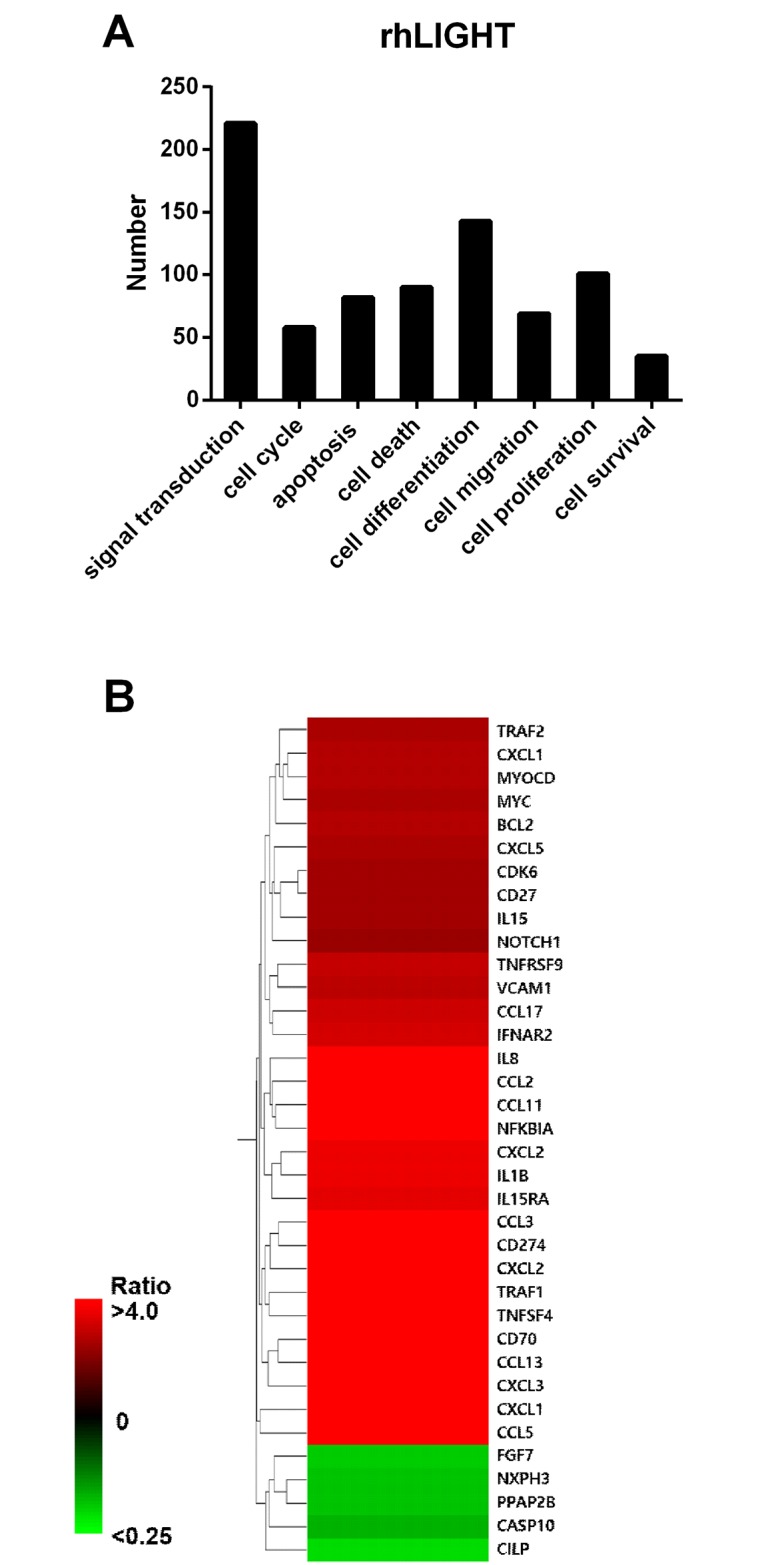
RhLIGHT activates various genes associated with TNF and chemokines in human BM-MSCs. Cells were incubated with 0, 100, and 200 ng/ml rhLIGHT for 48 h. (A) The number of genes in categorized pathways affected by rhLIGHT. (B) Microarray analysis of rhLIGHT-treated cells.

According to microarray results, many genes were altered by rhLIGHT in BM-MSCs, especially, those involved in signal transduction, cell differentiation, and cell proliferation (Fig 6A). rhLIGHT upregulated TNF genes, namely, the genes encoding TNFSF4 (OX40L), TNFRSF7 (CD27) TNFSF7 (CD70), CD274, and TNFRSF9 (4-1BB). In addition, rhLIGHT induced the expression of genes encoding diverse chemokines in BM-MSCs, such as CXCL1, CXCL2, CCL3, CCL5, CCL17, IL-1b, and IL-8. Moreover, the expression of survival genes, namely, BCL-2 and cell cycle-associated genes, MYC and CDK6, was increased by rhLIGHT in BM-MSCs (Fig 6B). In case of BCL-2, the expression of genes and proteins showed the same pattern in BM-MSCs (Figs 4B and 6B).

## Discussion

In the past decade, many basic studies showed the brilliant results of MSC-based therapeutic plans, including myocardial infarcts [32], diabetes [33], neurological disorders [5,7], and GVHD [7,12]. In addition, MSCs exhibit therapeutic potential for diverse diseases, including *Osteogenesis imperfecta* [10], GVHD [11–13], myocardial infarction [14,15], Crohn’s disease [16], alcoholic cirrhosis [17], and amyotrophic lateral sclerosis [18,19]. Thus, many reports indicate that these treatments are very effective and offer therapeutic promises for several diseases. However, only low number of cells (1–10 of 1 × 10^5^ nucleated cells) were collected from healthy volunteers by BM aspiration [7]. Thus, clinical application has always been limited because of such issues. Therefore, it is necessary to search for solutions to these problems. The most important approach could be the modification of MSCs before transplantation. This has developed into a promising option for enhancing the beneficial effects of MSC-based therapy. For example, modification of MSCs has helped cardiac tissue repair after myocardial infarction [36]. Therefore, we hypothesized that TNF and TNFR interaction play a significant role in the immune system and that they might be very effective in the modification of MSCs before transplantation.

LIGHT is a member of the TNF superfamily, and has three receptors, namely, HVEM, LTβR, and DcR3 [23,26]. These receptors, with distinct cellular expression patterns, are described to interact with LIGHT [24–26]. LIGHT activates T cell response in the immune system via HVEM [26,28]. LTβR is famous for its contribution toward the organization of lymphoid architecture and cellular positioning by other ligand, the lymphotoxin α/β [25]. However, the effects of LIGHT and LTβR interaction in human BM-MSCs are unclear.

It has been well known that pluripotency of the MSCs derived from the adult BM is the best [3]. Therefore, we used BM-derived MSCs in our study. We confirmed the quality of BM-derived MSCs, and analyzed them using FACS analysis to study cell surface molecule expression [2,5,6]. The phenotype of BM-MSCs was confirmed, including the negative (CD34, CD45, and CD19) and positive markers (CD90, CD44, and CD105; Fig 1A and 1B). Second, we also confirmed the differentiation quality by staining for adipogenesis (Oil Red O staining), chondrogenesis (Alcian blue staining), and osteogenesis (Alizarin red staining), as shown in Fig 1C. We screened the receptors of LIGHT in BM-MSCs. Human BM-MSCs expressed LTβR on the cell surface, not HVEM as expected (Fig 2A). HVEM was expressed on the neutrophils and monocytes from peripheral blood (Fig 2B). Then, we monitored the effects of rhLIGHT on human BM-MSCs. After rhLIGHT treatment, augmented cell numbers (Fig 3), cell viability (Fig 4A), cell survival, anti-apoptotic proteins (Fig 4B), cell proliferation (Fig 4C), and cell cycle progression (Fig 4D and 4E) were observed. Moreover, it induced MSC proliferation by increasing the S/G2/M phase. At the same time, cyclins and CDKs were increased, and CDKI p27 was decreased by rhLIGHT treatment Also, the production of PDGF and TGFβ was enhanced by rhLIGHT, but this depended on STAT3 and Smad3 activation (Fig 5). RhLIGHT upregulates the genes encoding TNF and chemokines in BM-MSCs (Fig 6). Thus, LIGHT (TNFSF14) obviously increases the survival and proliferation of human BM-MSCs via LTβR, not HVEM.

In addition, we were interested in the effects of rhLIGHT on differentiation quality, including the effects on adipogenesis, chondrogenesis, and osteogenesis. We followed the schedule shown in S1 Fig, plan B. We found that rhLIGHT treatment of human BM-MSCs did not have any effect on the differentiation quality (S1 Fig) and positive markers (S2 Fig). These results indicated that the property of human BM-MSCs was maintained despite rhLIGHT treatment. RhLIGHT enhances the differentiation quality of BM-MSCs (Chondrogenesis < Adipogenesis < Osteogenesis), as shown in S1 Fig. Liu et al. has already shown us that LIGHT/LTβR regulated the adipogenesis of BM-MSCs in mouse system, suggesting that recombinant mouse LIGHT controls the BM niche [37]. Therefore, we think that LIGHT can be used in stem cell therapy for modification of MSCs.

Several physiological agents, such as chemokines, cytokines and growth factors have been shown to induce ectodomain shedding [38]. Moreover, ectodomain shedding controls the activity of a number of transmembrane proteins. TNFSF and TNFRSF proteins have also been shown to be regulated by ectodomain shedding. For example, TNF-α [39], TGF-α [40], and HVEM [41,42]. They release the receptor-binding domain into the extracellular space [39,40,42]. It has been recently reported that LIGHT inhibits osteoblastogenesis of MSC co-cultured with monocytes in multiple myeloma-bone disease [43]. There are possibilities that LIGHT signaling might be hampered by various cytokines or factors produced in the environment including soluble LIGHT, soluble HVEM, and its soluble receptor, DcR3.

**Fig 7:**
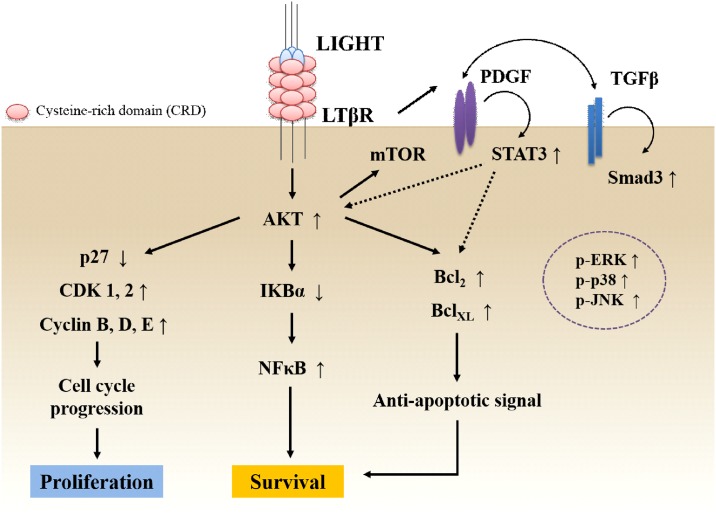
Proposed pathway of LIGHT and LTβR interaction in human BM-MSCs. LIGHT and LTβR interaction increases the survival and proliferation of human BM-MSCs by activating survival proteins, anti-apoptotic proteins, CDKs, and cyclins. Moreover, LIGHT-induced STAT-3 and smad-3 activation causes PDGF and TGF-β production, and they enhance LIGHT signals in human BM-MSCs. Therefore, LIGHT may play an important role in stem cell therapy, and contribute to MSC modification.

In conclusion, LIGHT and LTβR interaction increases the survival and proliferation of human BM-MSCs by activation of survival proteins, anti-apoptotic proteins, CDKs, and cyclins. Moreover, LIGHT-induced STAT-3 and smad-3 activation induces the production of PDGF and TGF-β, and enhances LIGHT signals in human BM-MSCs. We proposed the pathway of LIGHT and LTβR interaction in human BM-MSCs, as shown in Fig 7. Therefore, LIGHT may play an important role in stem cell therapy involving stem cells, and contribute to the modification of MSCs.

## Supporting Information

S1 Fig RhLIGHT does not affect differentiation quality. Cells were incubated with 0, 100, and 200 ng/mL rhLIGHT for 72 h. (A) Schedule of rhLIGHT treatment and staining cells undergoing adipogenesis (Oil Red O staining), chondrogenesis (Alcian blue staining), and osteogenesis (Alizarin red staining). (B) Images of adipocytes, chondrocytes, and osteocytes subjected to BSA-control (0.1% BSA-PBS buffer, upper panel) and rhLIGHT treatment (lower panel) in human BM-MSCs.
(TIF)

S2 Fig RhLIGHT does not affect the positive markers in human BM-MSCs. Cells were incubated with 0, 100, and 200 ng/mL rhLIGHT for 72 h. (A) Staining for the positive marker CD44 in BM-MSCs. (B) Staining for the positive marker CD90 in BM-MSCs. The expression of each marker was determined by FACS analysis. Filled histogram represents the isotype control (mouse IgG), filled purple histogram represents each antigen on BSA-control treatment, and open green histogram represents each antigen after rhLIGHT treatment. Data represent the mean ± SEM. n.s., not significant; BSA, bovine serum albumin.
(TIF)

## Abbreviations

TNF: tumor necrosis factor
HVEM: herpes virus entry mediator
LTβR: LT-β receptor
BMCs: bone marrow cells
MSC: mesenchymal stem cells
BM-MSCs: bone marrow-derived mesenchymal stem cells

## References

[1] Pittenger MF, Mackay AM, Beck SC, Jaiswal RK, Douglas R, Mosca JD, et al (1999) Multilineage potential of adult human mesenchymal stem cells. Science 284: 143–147. 10102814 10.1126/science.284.5411.143

[2] Gnecchi M, Melo LG (2009) Bone marrow-derived mesenchymal stem cells: isolation, expansion, characterization, viral transduction, and production of conditioned medium. Methods Mol Biol 482: 281–294. 10.1007/978-1-59745-060-7_18 19089363

[3] Jiang Y, Jahagirdar BN, Reinhardt RL, Schwartz RE, Keene CD, Ortiz-Gonzalez XR, et al (2002) Pluripotency of mesenchymal stem cells derived from adult marrow. Nature 418: 41–49. 10.1038/nature00870 12077603

[4] De Miguel MP, Fuentes-Julian S, Blazquez-Martinez A, Pascual CY, Aller MA, Arias J, et al (2012) Immunosuppressive properties of mesenchymal stem cells: advances and applications. Curr Mol Med 12: 574–591. 22515979 10.2174/156652412800619950

[5] Phinney DG, Prockop DJ (2007) Concise review: mesenchymal stem/multipotent stromal cells: the state of transdifferentiation and modes of tissue repair--current views. Stem Cells 25: 2896–2902. 10.1634/stemcells.2007-0637 17901396

[6] Nicolay NH, Lopez Perez R, Debus J, Huber PE (2015) Mesenchymal stem cells—A new hope for radiotherapy-induced tissue damage? Cancer Lett 366: 133–140. 10.1016/j.canlet.2015.06.012 26166559

[7] Si YL, Zhao YL, Hao HJ, Fu XB, Han WD (2011) MSCs: Biological characteristics, clinical applications and their outstanding concerns. Ageing Res Rev 10: 93–103. 10.1016/j.arr.2010.08.005 20727988

[8] Nauta AJ, Fibbe WE (2007) Immunomodulatory properties of mesenchymal stromal cells. Blood 110: 3499–3506. 10.1182/blood-2007-02-069716 17664353

[9] Najar M, Raicevic G, Crompot E, Fayyad-Kazan H, Bron D, Toungouz M, et al (2016) The Immunomodulatory Potential of Mesenchymal Stromal Cells: A Story of a Regulatory Network. J Immunother 39: 45–59. 10.1097/CJI.0000000000000108 26849074

[10] Horwitz EM, Gordon PL, Koo WK, Marx JC, Neel MD, McNall RY, et al (2002) Isolated allogeneic bone marrow-derived mesenchymal cells engraft and stimulate growth in children with osteogenesis imperfecta: Implications for cell therapy of bone. Proc Natl Acad Sci U S A 99: 8932–8937. 10.1073/pnas.132252399 12084934 PMC124401

[11] Le Blanc K, Frassoni F, Ball L, Locatelli F, Roelofs H, Lewis I, et al (2008) Mesenchymal stem cells for treatment of steroid-resistant, severe, acute graft-versus-host disease: a phase II study. Lancet 371: 1579–1586. 10.1016/S0140-6736(08)60690-X 18468541

[12] Koc ON, Lazarus HM (2001) Mesenchymal stem cells: heading into the clinic. Bone Marrow Transplant 27: 235–239. 10.1038/sj.bmt.1702791 11277170

[13] Le Blanc K, Rasmusson I, Sundberg B, Gotherstrom C, Hassan M, Uzunel M, et al (2004) Treatment of severe acute graft-versus-host disease with third party haploidentical mesenchymal stem cells. Lancet 363: 1439–1441. 10.1016/S0140-6736(04)16104-7 15121408

[14] Chen SL, Fang WW, Ye F, Liu YH, Qian J, Shan SJ, et al (2004) Effect on left ventricular function of intracoronary transplantation of autologous bone marrow mesenchymal stem cell in patients with acute myocardial infarction. Am J Cardiol 94: 92–95. 10.1016/j.amjcard.2004.03.034 15219514

[15] Stamm C, Westphal B, Kleine HD, Petzsch M, Kittner C, Klinge H, et al (2003) Autologous bone-marrow stem-cell transplantation for myocardial regeneration. Lancet 361: 45–46. 10.1016/S0140-6736(03)12110-1 12517467

[16] Duijvestein M, Vos AC, Roelofs H, Wildenberg ME, Wendrich BB, Verspaget HW, et al (2010) Autologous bone marrow-derived mesenchymal stromal cell treatment for refractory luminal Crohn's disease: results of a phase I study. Gut 59: 1662–1669. 10.1136/gut.2010.215152 20921206

[17] Suk KT, Yoon JH, Kim MY, Kim CW, Kim JK, Park H, et al (2016) Transplantation with Autologous Bone Marrow-Derived Mesenchymal Stem Cells for Alcoholic Cirrhosis: Phase 2 Trial. Hepatology. 10.1002/hep.28693 27339398

[18] Mazzini L, Fagioli F, Boccaletti R, Mareschi K, Oliveri G, Olivieri C, et al (2003) Stem cell therapy in amyotrophic lateral sclerosis: a methodological approach in humans. Amyotroph Lateral Scler Other Motor Neuron Disord 4: 158–161. 13129802 10.1080/14660820310014653

[19] Martinez HR, Molina-Lopez JF, Gonzalez-Garza MT, Moreno-Cuevas JE, Caro-Osorio E, Gil-Valadez A, et al (2012) Stem cell transplantation in amyotrophic lateral sclerosis patients: methodological approach, safety, and feasibility. Cell Transplant 21: 1899–1907. 10.3727/096368911X582769 23356668

[20] Locksley RM, Killeen N, Lenardo MJ (2001) The TNF and TNF receptor superfamilies: integrating mammalian biology. Cell 104: 487–501. 11239407 10.1016/s0092-8674(01)00237-9

[21] Chen L, Flies DB (2013) Molecular mechanisms of T cell co-stimulation and co-inhibition. Nat Rev Immunol 13: 227–242. 10.1038/nri3405 23470321 PMC3786574

[22] Mauri DN, Ebner R, Montgomery RI, Kochel KD, Cheung TC, Yu GL, et al (1998) LIGHT, a new member of the TNF superfamily, and lymphotoxin alpha are ligands for herpesvirus entry mediator. Immunity 8: 21–30. 9462508 10.1016/s1074-7613(00)80455-0

[23] Wang J, Fu YX (2004) The role of LIGHT in T cell-mediated immunity. Immunol Res 30: 201–214. 10.1385/IR:30:2:201 15477661

[24] Gommerman JL, Browning JL (2003) Lymphotoxin/light, lymphoid microenvironments and autoimmune disease. Nat Rev Immunol 3: 642–655. 10.1038/nri1151 12974479

[25] Lu TT, Browning JL (2014) Role of the Lymphotoxin/LIGHT System in the Development and Maintenance of Reticular Networks and Vasculature in Lymphoid Tissues. Front Immunol 5: 47 10.3389/fimmu.2014.00047 24575096 PMC3920476

[26] Murphy KM, Nelson CA, Sedy JR (2006) Balancing co-stimulation and inhibition with BTLA and HVEM. Nat Rev Immunol 6: 671–681. 10.1038/nri1917 16932752

[27] Heo SK, Ju SA, Lee SC, Park SM, Choe SY, Kwon B, et al (2006) LIGHT enhances the bactericidal activity of human monocytes and neutrophils via HVEM. J Leukoc Biol 79: 330–338. 10.1189/jlb.1104694 16275888

[28] Tamada K, Shimozaki K, Chapoval AI, Zhu G, Sica G, Flies D, et al (2000) Modulation of T-cell-mediated immunity in tumor and graft-versus-host disease models through the LIGHT co-stimulatory pathway. Nat Med 6: 283–289. 10.1038/73136 10700230

[29] Lin WW, Hsieh SL (2011) Decoy receptor 3: a pleiotropic immunomodulator and biomarker for inflammatory diseases, autoimmune diseases and cancer. Biochem Pharmacol 81: 838–847. 10.1016/j.bcp.2011.01.011 21295012

[30] Colucci S, Brunetti G, Mori G, Oranger A, Centonze M, Mori C, et al (2009) Soluble decoy receptor 3 modulates the survival and formation of osteoclasts from multiple myeloma bone disease patients. Leukemia 23: 2139–2146. 10.1038/leu.2009.136 19587706

[31] Chang PM, Chen PM, Hsieh SL, Tzeng CH, Liu JH, Chiou TJ, et al (2008) Expression of a soluble decoy receptor 3 in patients with diffuse large B-cell lymphoma predicts clinical outcome. Int J Oncol 33: 549–554. 18695885

[32] Bamias G, Gizis M, Delladetsima I, Laoudi E, Siakavellas SI, Koutsounas I, et al (2016) Elevated Serum Levels of the Antiapoptotic Protein Decoy-Receptor 3 Are Associated with Advanced Liver Disease. Can J Gastroenterol Hepatol 2016: 2637010 10.1155/2016/2637010 27595094 PMC4993922

[33] van der Garde M, van Pel M, Millan Rivero JE, de Graaf-Dijkstra A, Slot MC, Kleinveld Y, et al (2015) Direct Comparison of Wharton's Jelly and Bone Marrow-Derived Mesenchymal Stromal Cells to Enhance Engraftment of Cord Blood CD34(+) Transplants. Stem Cells Dev 24: 2649–2659. 10.1089/scd.2015.0138 26414086 PMC4652197

[34] Heo SK, Noh EK, Yoon DJ, Jo JC, Choi Y, Koh S, et al (2015) Radotinib Induces Apoptosis of CD11b+ Cells Differentiated from Acute Myeloid Leukemia Cells. PLoS One 10: e0129853 10.1371/journal.pone.0129853 26065685 PMC4466365

[35] Ng F, Boucher S, Koh S, Sastry KS, Chase L, Lakshmipathy U, et al (2008) PDGF, TGF-beta, and FGF signaling is important for differentiation and growth of mesenchymal stem cells (MSCs): transcriptional profiling can identify markers and signaling pathways important in differentiation of MSCs into adipogenic, chondrogenic, and osteogenic lineages. Blood 112: 295–307. 10.1182/blood-2007-07-103697 18332228

[36] Song H, Song BW, Cha MJ, Choi IG, Hwang KC (2010) Modification of mesenchymal stem cells for cardiac regeneration. Expert Opin Biol Ther 10: 309–319. 10.1517/14712590903455997 20132054

[37] Liu C, Ding H, Zhu W, Jiang S, Xu J, Zou GM (2013) LIGHT regulates the adipogenic differentiation of mesenchymal stem cells. J Cell Biochem 114: 346–353. 10.1002/jcb.24369 22930663

[38] Arribas J, Borroto A (2002) Protein ectodomain shedding. Chem Rev 102: 4627–4638. 12475204 10.1021/cr010202t

[39] Hikita A, Tanaka N, Yamane S, Ikeda Y, Furukawa H, Tohma S, et al (2009) Involvement of a disintegrin and metalloproteinase 10 and 17 in shedding of tumor necrosis factor-alpha. Biochem Cell Biol 87: 581–593. 10.1139/o09-015 19767822

[40] Wong ST, Winchell LF, McCune BK, Earp HS, Teixido J, Massague J, et al (1989) The TGF-alpha precursor expressed on the cell surface binds to the EGF receptor on adjacent cells, leading to signal transduction. Cell 56: 495–506. 2464440 10.1016/0092-8674(89)90252-3

[41] Jung HW, La SJ, Kim JY, Heo SK, Kim JY, Wang S, et al (2003) High levels of soluble herpes virus entry mediator in sera of patients with allergic and autoimmune diseases. Exp Mol Med 35: 501–508. 10.1038/emm.2003.65 14749527

[42] Heo SK, Ju SA, Kim GY, Park SM, Back SH, Park NH, et al (2012) The presence of high level soluble herpes virus entry mediator in sera of gastric cancer patients. Exp Mol Med 44: 149–158. 10.3858/emm.2012.44.2.010 22113134 PMC3296811

[43] Brunetti G, Rizzi R, Oranger A, Gigante I, Mori G, Taurino G, et al (2014) LIGHT/TNFSF14 increases osteoclastogenesis and decreases osteoblastogenesis in multiple myeloma-bone disease. Oncotarget 5: 12950–12967. 10.18632/oncotarget.2633 25460501 PMC4350341

