# Supplementary figures and images for: LIGHT (TNFSF14) Increases the Survival and Proliferation of Human Bone Marrow-Derived Mesenchymal Stem Cells
Source: PLoS One. 2016 Nov 11;11(11):e0166589. doi: 10.1371/journal.pone.0166589 (PMC5106019; doi:10.1371/journal.pone.0166589)

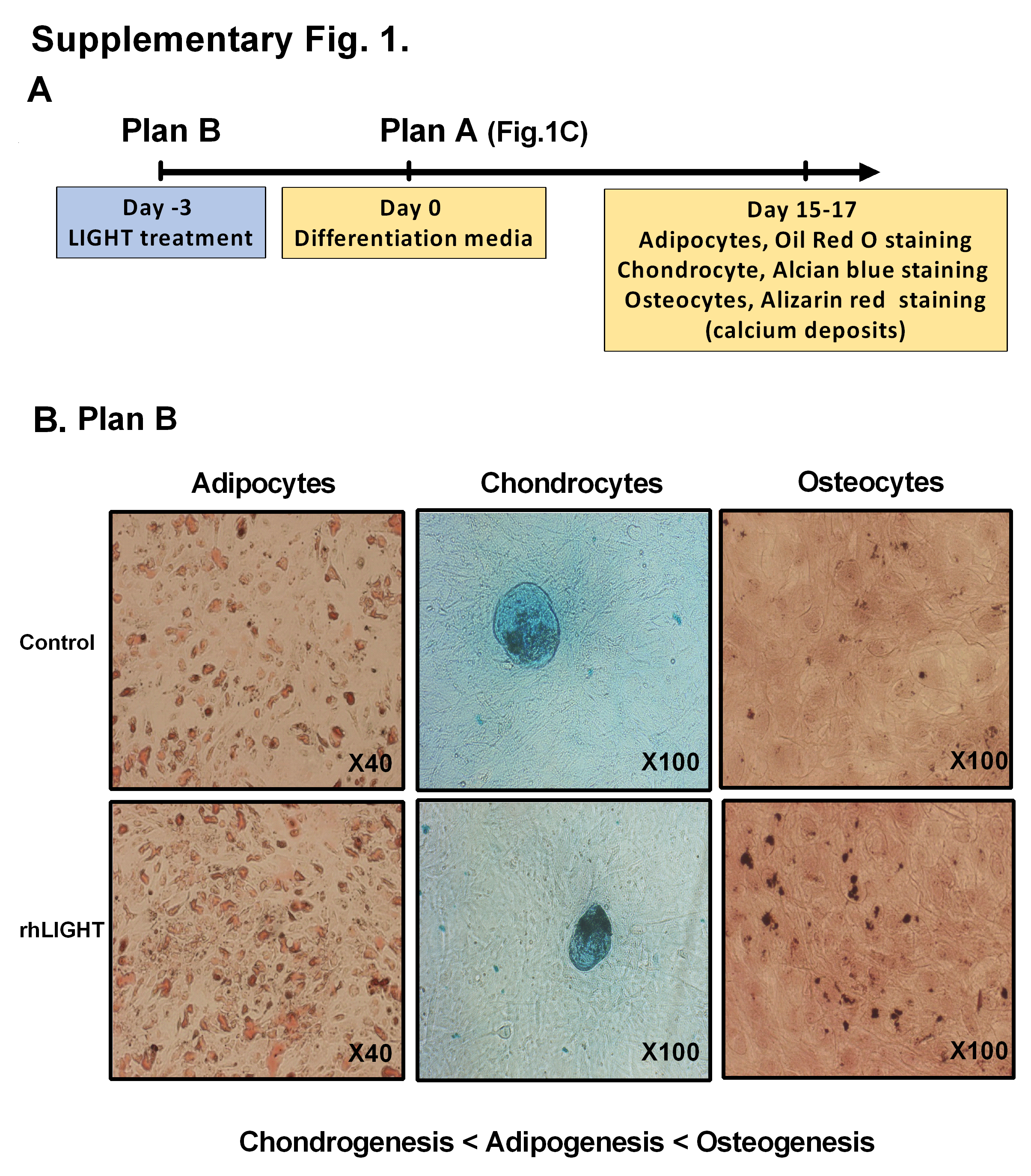

Supplement: S1 Fig — Cells were incubated with 0, 100, and 200 ng/mL rhLIGHT for 72 h. (A) Schedule of rhLIGHT treatment and staining cells undergoing adipogenesis (Oil Red O staining), chondrogenesis (Alcian blue staining), and osteogenesis (Alizarin red staining). (B) Images of adipocytes, chondrocytes, and osteocytes subjected to BSA-control (0.1% BSA-PBS buffer, upper panel) and rhLIGHT treatment (lower panel) in human BM-MSCs. (TIF) [file pone.0166589.s001.tif]

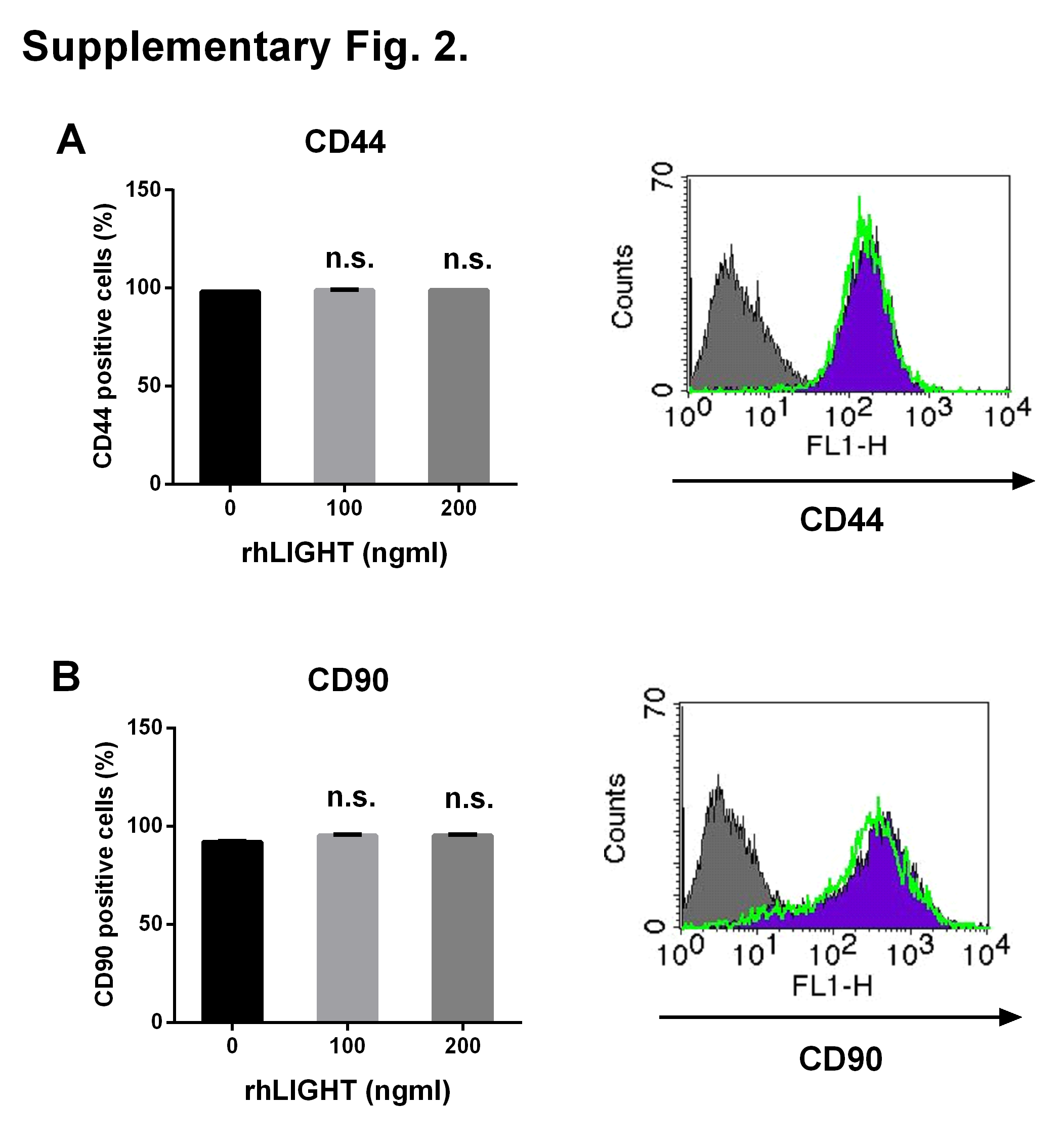

Supplement: S2 Fig — Cells were incubated with 0, 100, and 200 ng/mL rhLIGHT for 72 h. (A) Staining for the positive marker CD44 in BM-MSCs. (B) Staining for the positive marker CD90 in BM-MSCs. The expression of each marker was determined by FACS analysis. Filled histogram represents the isotype control (mouse IgG), filled purple histogram represents each antigen on BSA-control treatment, and open green histogram represents each antigen after rhLIGHT treatment. Data represent the mean ± SEM. n.s., not significant; BSA, bovine serum albumin. (TIF) [file pone.0166589.s002.tif]
